# 4D flow cardiovascular magnetic resonance for monitoring of aortic valve repair in bicuspid aortic valve disease

**DOI:** 10.1186/s12968-020-00608-0

**Published:** 2020-04-30

**Authors:** Alexander Lenz, Johannes Petersen, Christoph Riedel, Julius M. Weinrich, Hendrik Kooijman, Bjoern P. Schoennagel, Gerhard Adam, Yskert von Kodolitsch, Hermann Reichenspurner, Evaldas Girdauskas, Peter Bannas

**Affiliations:** 1 Department of Diagnostic and Interventional Radiology and Nuclear Medicine, University Medical Center Hamburg-Eppendorf, Martinistraße 52, 20246 Hamburg, Germany; 2 Department of Cardiovascular Surgery, University Heart Center, Hamburg, Germany; 3 Clinical Science Department, Philips GmbH, Hamburg, Germany; 4 Department of Cardiology, University Heart Center, Hamburg, Germany

**Keywords:** 4D flow MRI, Congenital heart disease, Bicuspid aortopathy, Adult congenital heart disease, Aortic valve repair, Aorta, Hemodynamics, Aortic regurgitation

## Abstract

**Background:**

Aortic valve repair has become a treatment option for adults with symptomatic bicuspid (BAV) or unicuspid (UAV) aortic valve insufficiency. Our aim was to demonstrate the feasibility of 4D flow cardiovascular magnetic resonance (CMR) to assess the impact of aortic valve repair on changes in blood flow dynamics in patients with symptomatic BAV or UAV.

**Methods:**

Twenty patients with adult congenital heart disease (median 35 years, range 18–64; 16 male) and symptomatic aortic valve regurgitation (15 BAV, 5 UAV) were prospectively studied. All patients underwent 4D flow CMR before and after aortic valve repair. Aortic valve regurgitant fraction and systolic peak velocity were estimated. The degree of helical and vortical flow was evaluated according to a 3-point scale. Relative flow displacement and wall shear stress (WSS) were quantified at predefined levels in the thoracic aorta.

**Results:**

All patients underwent successful aortic valve repair with a significant reduction of aortic valve regurgitation (16.7 ± 9.8% to 6.4 ± 4.4%, *p* < 0.001) and systolic peak velocity (2.3 ± 0.9 to 1.9 ± 0.4 m/s, *p* = 0.014). Both helical flow (1.6 ± 0.6 vs. 0.9 ± 0.5, *p* < 0.001) and vortical flow (1.2 ± 0.8 vs. 0.5 ± 0.6, *p* = 0.002) as well as both flow displacement (0.3 ± 0.1 vs. 0.25 ± 0.1, *p* = 0.031) and WSS (0.8 ± 0.2 N/m^2^ vs. 0.5 ± 0.2 N/m^2^, *p* < 0.001) in the ascending aorta were significantly reduced after aortic valve repair.

**Conclusions:**

4D flow CMR allows assessment of the impact of aortic valve repair on changes in blood flow dynamics in patients with bicuspid aortic valve disease.

## Background

Bicuspid (BAV) and unicuspid (UAV) aortic valve malformations represent two forms of adult congenital heart disease with a prevalence of 1–2 and 0.02%, respectively [1–4]. BAV and UAV are associated with aortic valve dysfunction (regurgitation and/or stenosis), dissection, and proximal aortic dilatation, the so-called bicuspid aortopathy [5]. Genetic and hemodynamic factors contribute to the progression of BAV and UAV disease [6, 7] and play a role in the development of bicuspid aortopathy, including the increased risk for aortic dissection [5, 8].

**Fig. 1:**
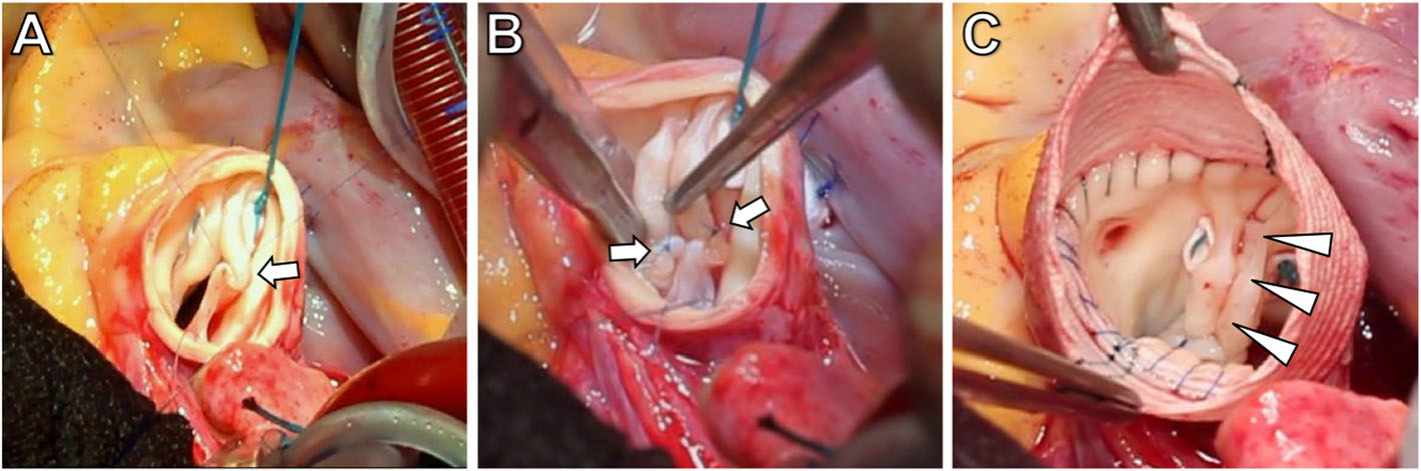
Intraoperative situs of aortic valve repair in bicuspid aortic valve (BAV) disease. The surgical repair consists of reduction of the aortic valve anulus using suture annuloplasty and correction of the prolapse of the fused cup. The aim is the recreation of the optimal aortic root geometry. This includes reduction of the basal ring diameter to less than 25 mm and restoration of effective cusp height (coaptation length) above 8 mm. **a** and **b** Correction of cusp prolapse by means of plication sutures (arrows). **c** Surgery results in a symmetric configuration of the bicuspid aortic valve (arrow heads) with a commissural angle of 180°, resembling a Sievers type 0 valve. In this patient, additional replacement of the aortic root with Dacron prosthesis for aneurysm has been performed

Surgical treatment for regurgitation and/or stenosis, particularly aortic valve repair techniques, underwent major development during the last decades. Surgical repair is a promising alternative to prosthetic aortic valve replacement, especially in young patients [2, 9–11]. Aortic valve repair has several advantages compared to aortic valve replacement, including absence of the need for chronic anticoagulation, lower infection rate, and better hemodynamic performance [12]. From a technical point of view, aortic valve repair is a two-component surgery, consisting of cusp repair and aortic valve annulus stabilization (Fig. 1) [2]. However, recurrent aortic valve regurgitation is still a major issue in patients after aortic valve repair as compared to those after surgical aortic valve replacement [12–14]. Therefore, the development of more durable aortic valve repair techniques remains an important clinical challenge. In this context, a comprehensive marker for accurate assessment of changes in hemodynamics after aortic valve repair is needed to evaluate surgical success.

Four-dimensional (4D)-flow cardiovascular magnetic resonance imaging (CMR) has been successfully used to visualize abnormal hemodynamic flow patterns such as helical and vortical flow [15–18], wall shear stress [19–23], and flow displacement (indicator of outflow asymmetry) [24–27] in untreated adult congenital heart disease and after aortic valve replacement surgery [28–31]. We hypothesize that 4D flow CMR might be a comprehensive tool to monitor aortic valve competence and hemodynamic changes after aortic valve repair. Therefore, the aim of this study was to demonstrate the feasibility of 4D flow CMR to assess the impact of aortic valve repair on changes in blood flow dynamics in adult congenital heart disease patients with symptomatic BAV or UAV.

## Methods

### Patients

This prospective study was approved by the local ethics board. Written informed consent was obtained from all patients.

Patients with adult congenital heart disease (BAV or UAV) and symptomatic, predominant aortic regurgitation who were referred for minimally invasive aortic valve repair between April 2017 and February 2019 were included in the study. Patients with contraindications for CMR or younger than 18 years were excluded.

Diagnosis of aortic regurgitation was based on transthoracic echocardiography. Echocardiography was also used to assess the quality of aortic cusp tissue and absence of severe calcifications prior to surgery. In case of intraoperative findings like severe cusp calcifications or fenestrations, aortic valve repair was not performed and patients were not included in this study. The procedure of aortic valve repair has been previously described [2].

### Cardiovascular magnetic resonance imaging

All patients underwent non-contrast 4D flow CMR of the thoracic aorta on a 3 T system (Ingenia, Philips Healthcare, Best, The Netherlands) with a 32-channel body-phased array coil before and after surgery. Respiratory gated and cardiac triggered 4D flow CMR data were acquired over the entire cardiac cycle with full volumetric coverage of the thoracic aorta. Scan parameters included: velocity encoding 200 cm/s, temporal resolution 24–38 ms, acquired spatial resolution 2.5 × 2.5 × 2.5 mm^3^, field of view (280–330) x (280–330) x (50–66) mm^3^, flip angle = 8°. Parallel imaging (k-t BLAST) with an acceleration factor of 4 was used. Scan time for each acquisition was 13.9 ± 3.0 min, depending on heart rate, respiratory pattern, and efficiency of respiratory gating.

Electrocardiogram (ECG)-gated CMR imaging was performed for assessment of left ventricular (LV) volumes and function by using balanced steady-state free-precession (bSSFP) cine CMR [32]. ECG-gated bSSFP imaging of the thoracic aorta was performed for assessment of aortic diameters [33, 34].

### 4D flow CMR data analysis

4D flow data were corrected for Maxwell terms, eddy currents and phase aliasing in accordance with current consensus recommendations [35]. All data sets were automatically reconstructed to 24 time frames per cardiac cycle and used to render three-dimensional phase-contrast CMR angiograms in a 3D visualization software (GTFlow, GyroTools LLC, Zurich, Switzerland).

One radiologist with 4 years of experience in the assessment of 4D flow data manually placed analysis planes at six defined anatomic landmarks in the thoracic aorta at the level of the aortic valve, the sinotubular junction, the mid-ascending aorta, the aortic arch proximal to the brachiocephalic trunc, the aortic arch distal to the left subclavian artery and the proximal descending aorta [36].

The same radiologist quantified peak velocity as well as forward and backward flow volumes at the aortic valve level and the regurgitant fraction (%) was calculated [37, 38].

Helical and vortical blood flow patterns in the ascending aorta (AAo), the aortic arch (AA), and the descending aorta (DAo) were semiquantitatively evaluated according to a 3-point scale: 0 (none), 1 (< 360°), and 2 (> 360°). A helical flow pattern was defined as a regional spiral movement along the blood flow direction and a vortical flow pattern as a regional circular movement deviating from the physiological flow-direction by > 90° [39, 40].

Flow displacement, a marker to quantify outflow asymmetry, was automatically quantified by exporting defined analyses planes into MATLAB (The MathWorks, Natick, Massachusetts, USA). Flow displacement was defined as the distance from the vessel centroid to the velocity-weighted centroid of the upper 15% of peak systolic forward flow velocity normalized to the vessel diameter, similar to the strategy reported by Sigovan et al. and Mahadevia et al [24, 26]

WSS, a time-resolved three-dimensional force, was quantified in GTFlow (Gyrotools). Magnitudinal WSS, which is the resulting net vector along the entire vascular wall, was derived from each analysis plane at peak systole [23, 41]. Values for peak systolic WSS were averaged over the five cardiac time frames centered on peak systole to reduce measurement noise [39]. Averaged circumferential WSS was assessed for each plane as well as segmental WSS at 8 standardized local anatomic orientations of the vessel wall: anterior (A), left-anterior (LA), left (L), left-posterior (LP), posterior (P), right-posterior (RP), right (R), and right-anterior (RA) [21, 26, 40].

### Statistical analysis

The D’Agostino-Pearson test was used to evaluate whether parameters were normally distributed. Data before and after surgery were compared by a two-sided paired t-test if normally distributed and by Wilcoxon matched-pairs signed-rank test if non-normally distributed. Patients were further divided into subgroups: group 1: BAV type 1 L/R; group 2: UAV. All *p* values < 0.05 were considered statistically significant. All continuous data are presented as mean ± standard deviation. To account for skewed data, median and interquartile ranges were calculated when appropriate. Statistical analyses were performed using GraphPad Prism 7 (GraphPad Software, San Diego, California, USA).

## Results

### Study sample

4D flow CMR before and after aortic valve repair was successfully completed in 20 patients with adult congenital heart disease and symptomatic aortic valve regurgitation. Three other patients had to be excluded from the main analyses. In one patient, the dilated aortic root was only partially covered by the 3D volume of the 4D flow sequence. Two patients required aortic valve replacement instead of repair and therefore did not undergo the second CMR examination.

Median age of the 20 included patients at the time of surgery was 35 years (IQR 29–47). Median time between CMR examinations was 9 days (IQR 6–38). There were no relevant medications changed between CMR imaging examinations.

**Fig. 2:**
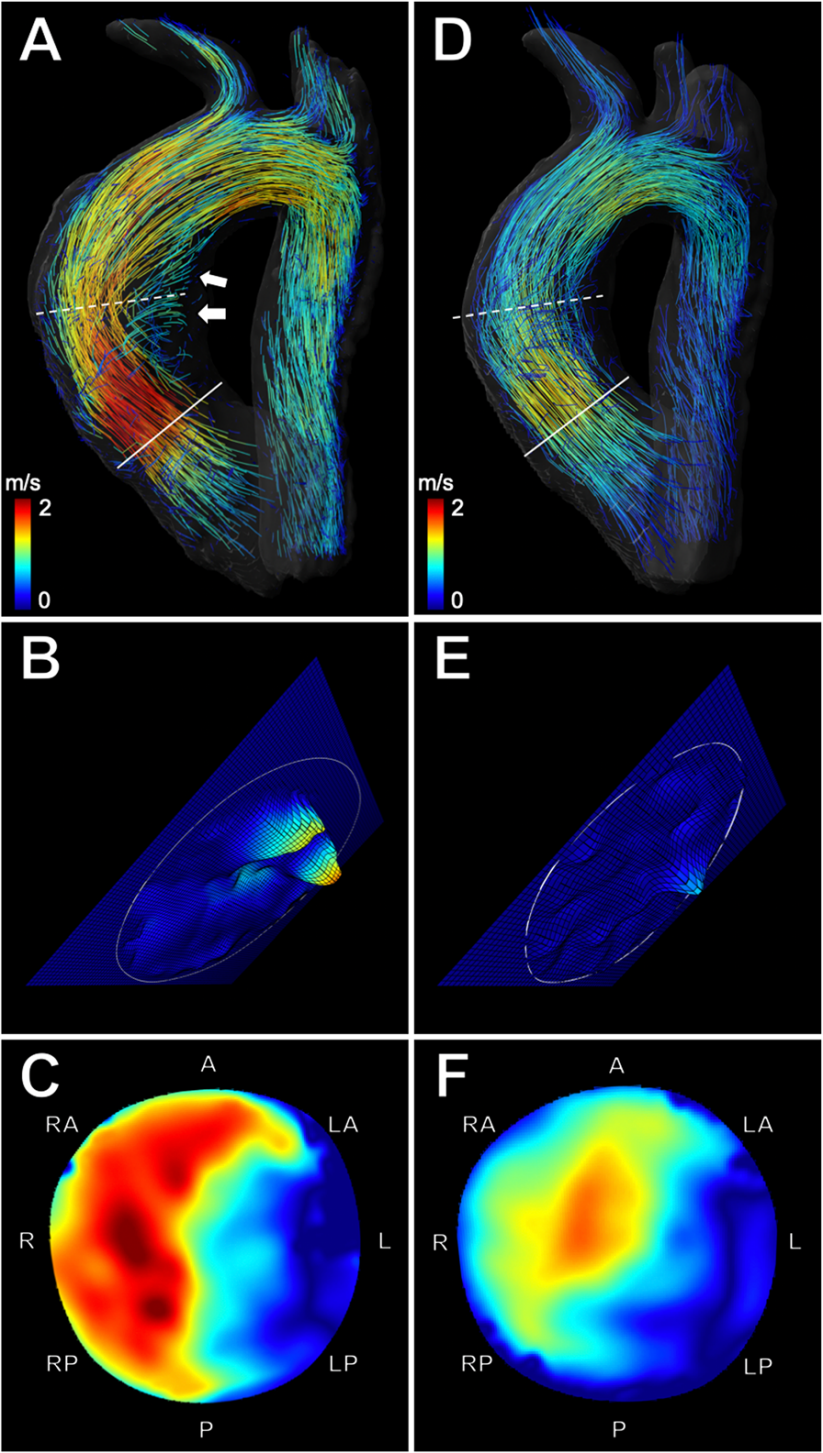
4D flow CMR-based characterization of flow dynamics in a 24-year-old man with bicuspid aortic valve before and after aortic valve repair. **a** Velocity-coded 4D flow CMR reveals an accelerated eccentric asymmetric flow jet (indicated by yellow and red streamlines) and a pronounced helical flow pattern (arrows) in the ascending aorta before surgery. The flow jet impacts and travels along the right aortic wall. **b** Extracted analysis plane (solid line) at the aortic valve level shows eccentric regurgitation of insufficient bicuspid valve (15.3%) (**c**) Extracted analysis plane (dashed line) at the level of the mid-ascending aorta shows the marked eccentric flow pattern (relative flow displacement: 0.43), resulting in increased global WSS (1.2 N/m^2^). **d** After surgery, velocity-coded 4D flow CMR shows reduced helical flow with a more cohesive central flow pattern more parallel to the vessel wall of the ascending aorta. **e** Extracted analysis plane at the aortic valve level after surgery shows decreased regurgitation (5.6%) (**f**) Extracted analysis plane at the level of the mid-ascending aorta demonstrates more centralized flow (relative flow displacement: 0.27), resulting in decreased global WSS (0.75 N/m^2^) after aortic valve repair

**Fig. 3:**
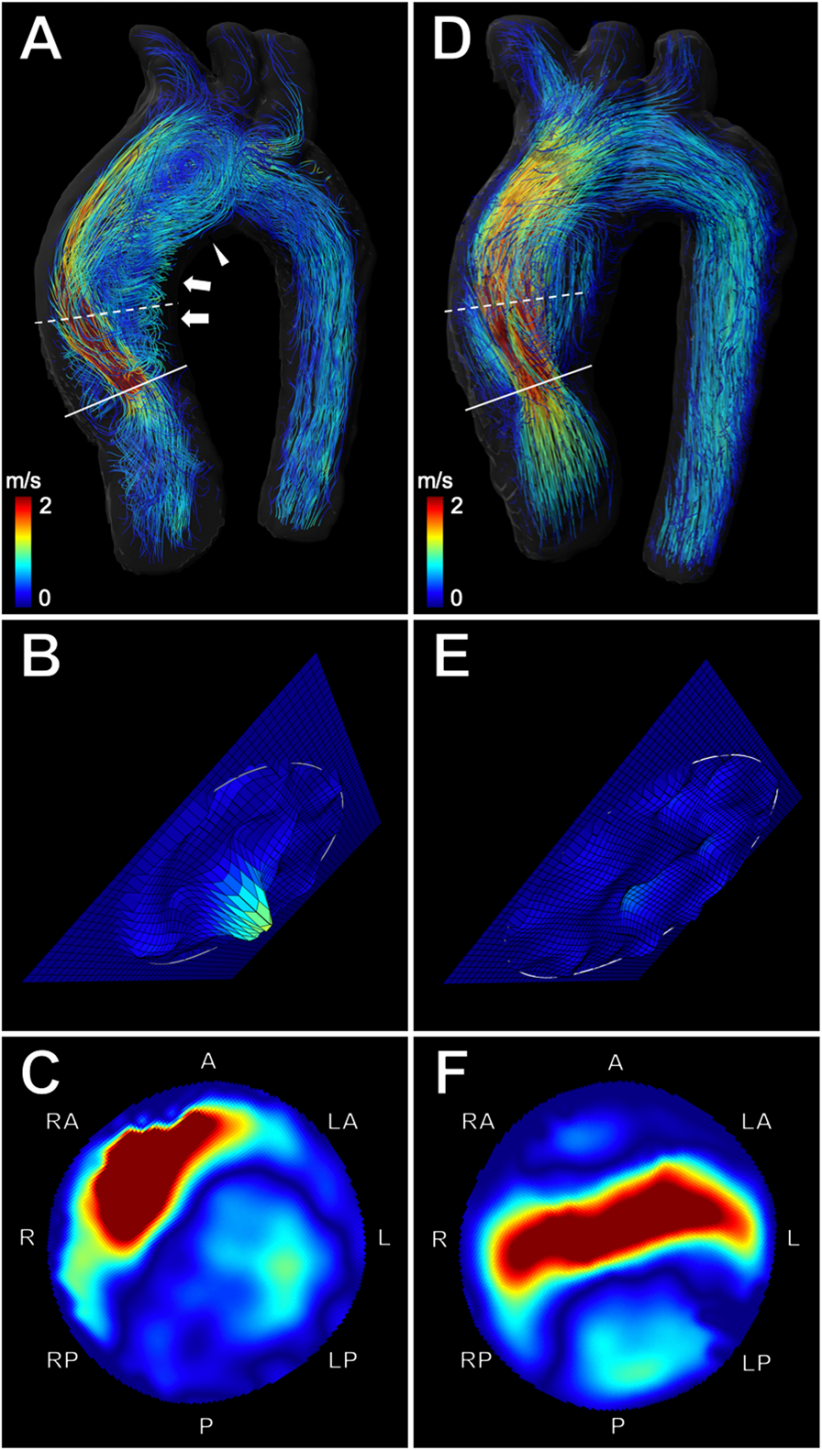
4D flow CMR-based characterization of flow dynamics in a 30-year-old woman with unicuspid aortic valve before and after aortic valve repair. **a** Velocity-coded 4D flow CMR reveals an accelerated and highly eccentric asymmetric flow jet (indicated by yellow and red streamlines) and a pronounced helical (arrows) and vortical flow pattern (arrowhead) in the ascending aorta before surgery. The flow jet impacts and travels along the right-anterior aortic wall. **b** Extracted analysis plane (solid line) at the aortic valve level shows regurgitation of insufficient bicuspid valve (20%) (**c**) Extracted analysis plane (dashed line) at the level of the mid-ascending aorta shows the marked eccentric flow pattern (relative flow displacement: 0.37), resulting in increased global WSS (0.9 N/m^2^). **d** After surgery, velocity-coded 4D flow CMR shows reduced helical and vortical flow with a more cohesive central flow pattern more parallel to the vessel wall of the ascending aorta. **e** Extracted analysis plane at the aortic valve level shows decreased regurgitation (5.8%) after surgery (**f**) Extracted analysis plane at the level of the mid-ascending aorta demonstrates a more centralized flow (relative flow displacement: 0.24), resulting in decreased global WSS (0.37 N/m^2^) after aortic valve repair

Fifteen patients (75%) had a BAV Sievers Type 1 phenotype with L/R cusp fusion **(**Fig. 2**)** and five patients (25%) presented with a UAV phenotype **(**Fig. 3) (Table 1). Seventeen patients (85%) underwent isolated aortic valve repair, while three patients (15%) needed additional aortic root remodeling [42]. After surgery, there was a significant reduction of mean aortic diameters at the level of the anulus (2.6 ± 0.4 vs. 2.3 ± 0.2 cm, *p* < 0.001), which is explained by suture annuloplasty aiming to reduce the basal ring diameter. The average diameter at the level of the bulbus aortae (3.8 ± 0.6 vs. 3.5 ± 0.4 cm, *p* = 0.03) and the mid-ascending aorta (3.3 ± 0.6 vs. 3.1 ± 0.4 cm, *p* = 0.03) was also significantly reduced, which is explained by the three patients with aortic root remodeling with Dacron prostheses. After surgery there was a significant reduction in stroke volume (120 ± 34 vs. 90 ± 28 ml, *p* < 0.001). There was no significant difference in LVEF after surgery (57.8 ± 7.8% vs. 58.7 ± 7.2%, *p* = 0.252). Patient characteristics of all patients and subgroup analyses of BAV and UAV patients including aortic diameters and cardiac function metrics before and after surgery are detailed in Table 1.

**Table 1:** Patient characteristics and aortic diameters before and after aortic valve repair

Characteristic	All patients ***n*** = 20			BAV (type L/R) ***n*** = 15			UAV ***n*** = 5		
Age at time of operation (y) *median*	35 (IQR 29-47)			42 (IQR 30-47)			30 (IQR 24-45)		
Gender, male	16			13			3		
Days between imaging *median*	9 (IQR 6-38)			7 (IQR 6-42)			13 (IQR 6-66)		
Procedures									
Isolated aortic valve repair	17			13			4		
Aortic root replacement (Yacoub)	3			2			1		
	**pre OP**	**post OP**	***P*value**	**pre OP**	**post OP**	***P*value**	**pre OP**	**post OP**	***P*value**
**Heart rate (bpm)**	77.8 ± 9.4	88.0 ± 14.4	**0.021**	79.0 ± 9.4	87.3 ± 12.1	ns	74.0 ± 9.3	90.2 ± 21.4	ns
**LV parameters**									
EDV (ml)	208.6 ± 71.9	149.1 ± 48.0	**<0.001**	223.1 ± 61.4	153.5 ± 36.9	**<0.001**	170.8 ± 81.9	157.0 ± 70.7	ns
ESV (ml)	86.3 ± 45.8	64.7 ± 28.2	**<0.001**	100.0 ± 39.6	68.1 ± 24.9	**<0.001**	56.8 ± 39.3	52.5 ± 39.5	ns
SV (ml)	119.6 ± 34.0	89.9 ± 27.9	**<0.001**	124.1 ± 32.9	88.4 ± 26.3	**<0.001**	98.3 ± 38.4	93.0 ± 36.4	ns
EF (%)	57.8 ± 7.8	58.7 ± 7.2	ns	56.3 ± 7.7	57.5 ± 7.2	ns	63.3 ± 6.6	61.4 ± 6.2	ns
**Dimensions (mm)**									
Anulus	26.3 ± 3.7	23.3 ± 2.4	**<0.001**	28.9 ± 3.6	23.4 ± 2.0	**<0.001**	24.8 ± 3.8	23.0 ± 3.4	**0.009**
Bulbus aortae	37.6 ± 5.5	35.2 ± 4.1	**0.031**	38.1 ± 6.0	35.0 ± 4.2	ns	36.0 ± 4.2	35.8 ± 4.1	ns
Mid-ascending aorta	33.1 ± 6.4	30.6 ± 4.1	**0.033**	33.4 ± 5.6	31.5 ± 4.2	**0.023**	32.2 ± 9.4	27.8 ± 2.4	ns
Proximal aortic arch	26.9 ± 4.0	26.2 ± 3.5	ns	27.5 ± 4.3	26.5 ± 3.7	ns	25.2 ± 2.8	25.0 ± 2.7	ns
Distal aortic arch	22.2 ± 2.7	22.1 ± 2.6	ns	22.8 ± 2.4	22.7 ± 2.4	ns	20.4 ± 2.8	20.4 ± 2.9	ns
Descending aorta	23.5 ± 4.0	23.2 ± 3.9	ns	24.0 ± 3.4	23.6 ± 3.4	ns	22.0 ± 5.6	22.0 ± 5.6	ns

### Quantification and visualization of blood flow

4D flow CMR-derived blood flow quantification revealed that aortic valve repair resulted in a significant reduction of forward flow, backward flow, and net flow volume at the aortic valve level (all *p* < 0.001) (Table 2). The improved aortic root geometry also resulted in a significantly reduced regurgitant fraction (17 ± 10% vs. 6 ± 4%, *p* < 0.001) (Fig. 2b and e, Fig. 3b and e) and systolic peak velocity (2.2 ± 0.9 vs. 1.9 ± 0.4 m/s, *p* = 0.014) (Table 2).

**Table 2:** Comparison of 4D flow CMR-derived flow volumes and flow patterns before and after aortic valve repair

Measure	pre OP	post OP	*p* value
Forward flow (ml)	120 ± 44	80 ± 24	**< 0.001**
Backward flow (ml)	21 ± 17	5 ± 3	**< 0.001**
Net flow (ml)	99 ± 36	75 ± 23	**< 0.001**
Peak systolic velocity (cm/s)	225 ± 85	192 ± 40	**0.014**
Regurgitant fraction (%)	17 ± 10	6 ± 4	**< 0.001**
Helix grade			
Ascending aorta	1.6 ± 0.6	0.9 ± 0.5	**< 0.001**
Aortic arch	0.5 ± 0.7	0.3 ± 0.5	ns
Descending aorta	0.3 ± 0.6	0.1 ± 0.4	ns
Vortex grade			
Ascending aorta	1.2 ± 0.8	0.5 ± 0.6	**0.002**
Aortic arch	0.2 ± 0.4	0.0 ± 0.0	ns
Descending aorta	0.3 ± 0.6	0.2 ± 0.4	ns

Values represent mean ± SD. *p* < 0.05 indicates a statistically significant difference. Significant values are in bold

4D flow CMR allowed visualization of hemodynamic flow patterns such as helical and vortical flow in the thoracic aorta before **(**Fig. 2a and Fig. 3a) and after aortic valve repair **(**Fig. 2d and Fig. 3d).

All patients showed common global right-handed helical flow formation in the AAo before and after surgery. Pathologic, secondary local helical flow formations in the AAo were reduced after surgery in 13 of 20 patients (65%) (1.6 ± 0.6 vs. 0.9 ± 0.5, *p* < 0.001). Helical flow in the aortic arch and DAo was less pronounced, however, these differences where not statistically significant **(**Table 2).

Vortical flow patterns in the AAo were observed in 15 of 20 patients (75%) before surgery and in 9 of 20 patients (45%) after surgery, resulting in a significant decrease of the average vortical flow (1.2 ± 0.8 vs. 0.5 ± 0.6, *p* = 0.002). Although vortical flow was less pronounced in the aortic arch and DAo after surgery, these differences were not statistically significant **(**Table 2).

### Flow displacement

**Fig. 4:**
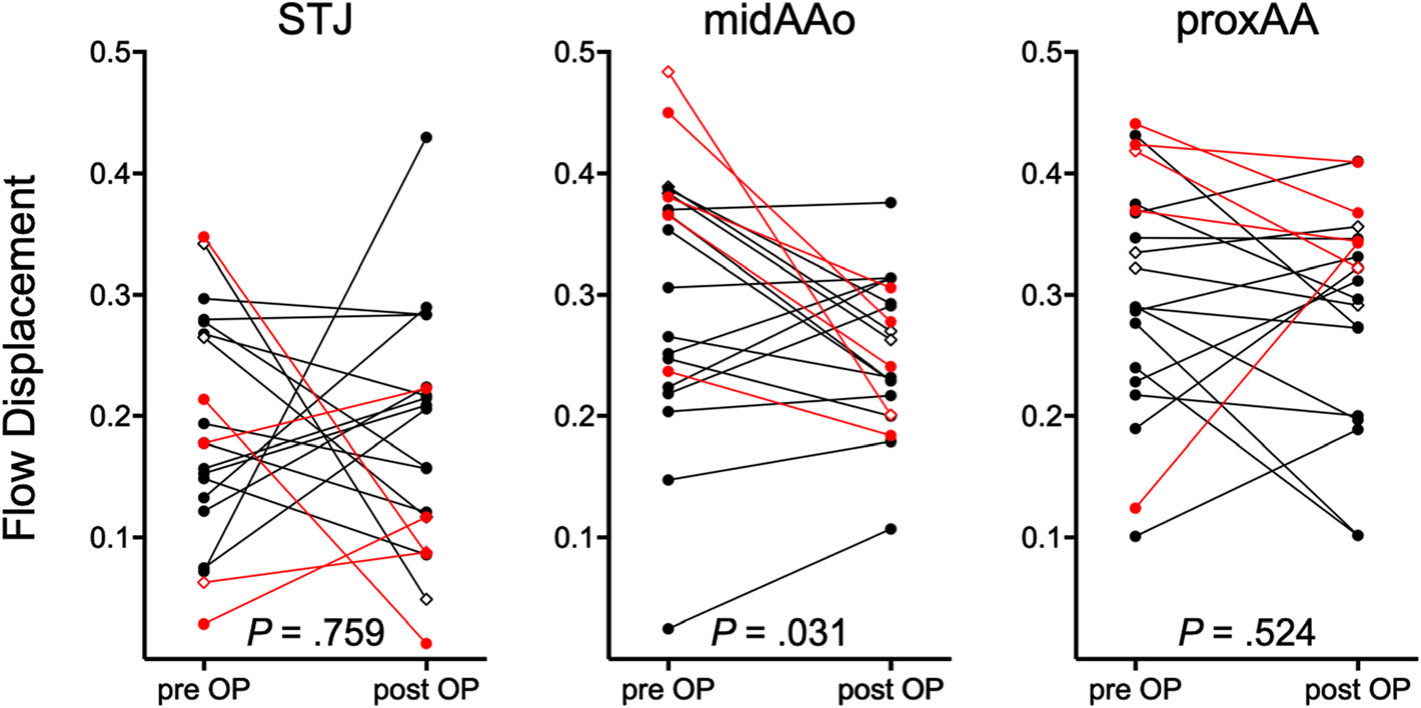
Flow displacement in the ascending aorta before and after aortic valve repair in patients with adult congenital heart disease. Flow displacement was significantly reduced after surgery (0.3 ± 0.1 vs. 0.25 ± 0.1) at the level of the mid-ascending aorta (midAAo). There was no significant reduction in flow displacement at the level of the sinotubular junction (STJ) and the proximal aortic arch (proxAA). Red lines indicate patients with unicuspid aortic valves and diamonds indicate patients with additional aortic root remodeling

4D flow CMR revealed highly eccentric outflow jet patterns directed towards the right-anterior wall of the mid-ascending AAo before surgery **(**Fig. 2c and Fig. 3c) in all patients and a more centrally located flow profile after aortic valve repair (Fig. 2f and Fig. 3f) in twelve patients (60%) (Fig. 4). Flow displacement was significantly reduced after surgery in the mid-ascending AAo (0.3 ± 0.1 vs. 0.25 ± 0.1, *p*= 0.031) (Fig. 4). There was no significant change of flow displacement at the level of the sinotubular junction (0.19 ± 0.1 vs. 0.18 ± 0.1, *p* = 0.759) and the proximal aortic arch (0.3 ± 0.1 vs. 0.29 ± 0.1, *p* = 0.524).

Subgroup analyses of BAV and UAV patients are detailed in Table 3.

**Table 3:** Flow displacement subgroups

Group	pre OP	post OP	***P*** value
**BAV**			
Sinutubular junction	0.20 ± 0.1	0.20 ± 0.1	ns
Mid-ascending aorta	0.28 ± 0.1	0.26 ± 0.1	ns
Proximal aortic arch	0.29 ± 0.0	0.27 ± 0.1	ns
**UAV**			
Sinutubular junction	0.17 ± 0.1	0.10 ± 0.1	ns
Mid-ascending aorta	0.38 ± 0.1	0.24 ± 0.1	**0.026**
Proximal aortic arch	0.19 ± 0.1	0.32 ± 0.4	ns

Values represent mean ± SD. *P* < 0.05 indicates a statistically significant difference. Significant values are in bold

### Circumferential aortic wall shear stress

**Fig. 5:**
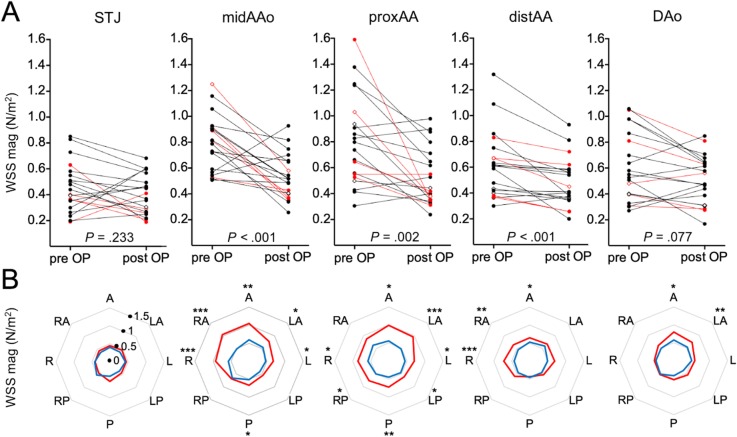
Circumferential and segmental wall shear stress (WSS) in the thoracic aorta before and after aortic valve repair in patients with congenital heart disease. **a** Graphs of quantitative analyses of circumferential peak systolic WSS show a significant reduction at the level of the mid-ascending aorta (midAAo), proximal aortic arch (proxAA), and distal aortic arch (distAA). Red lines indicate patients with unicuspid aortic valves and diamonds indicate patients with additional aortic root remodeling. **b** Spider charts of segmental peak systolic WSS at eight standardized local anatomic positions of the vessel wall (A, anterior; LA, left anterior; L, left; LP, left posterior; P, posterior; RP, right posterior; R, right; RA, right anterior) before (red spiders) and after (blue spiders) surgery. Highest segmental WSS was observed in the anterior, right-anterior, and right segments in the mid-ascending aorta as well as in the anterior and left-anterior segments of the proximal aortic arch. Asterisks indicate segments with significantly reduced WSS after aortic valve surgery. Of note, changes in peak systolic segmental WSS values are co-located with the changes in localized outflow jets and the position of elevated velocity before and after surgery

Circumferential WSS was significantly reduced after aortic valve repair in the mid-ascending AAo (0.8 ± 0.2 vs. 0.5 ± 0.2 N/m^2^, *p* < 0.001), proximal aortic arch (0.8 ± 0.4 vs. 0.5 ± 0.2 N/m^2^, *p* = 0.002), and distal aortic arch (0.6 ± 0.3 vs. 0.5 ± 0.2 N/m^2^, *P* < 0.001) for all patients (Fig. 5a). There was no significant change at the level of the sinotubular junction (0.5 ± 0.2 vs. 0.4 ± 0.2 N/m^2^, *p* = 0.233) and the DAo (0.6 ± 0.3 vs. 0.5 ± 0.2 N/m^2^, *p* = 0.077). In the mid-ascending AAo, where circumferential WSS was highest, 4D flow CMR revealed decreased WSS postoperatively in 18 of 20 patients (90%) and an increased WSS in two patients (10%).

Subgroup analyses of BAV and UAV patients are detailed in Table 4.

**Table 4:** Circumferential aortic wall shear stress subgroups

Group	pre OP	post OP	***P*** value
**BAV**			
Sinutubular junction (N/m^2^)	0.5 ± 0.2	0.4 ± 0.1	ns
Mid-ascending aorta (N/m^2^)	0.8 ± 0.2	0.6 ± 0.2	**0.009**
Proximal aortic arch (N/m^2^)	0.8 ± 0.3	0.6 ± 0.2	**0.006**
Distal aortic arch (N/m^2^)	0.6 ± 0.3	0.5 ± 0.2	**0.003**
Descending aorta (N/m^2^)	0.6 ± 0.3	0.5 ± 0.2	ns
**UAV**			
Sinutubular junction (N/m^2^)	0.4 ± 0.2	0.3 ± 0.1	ns
Mid-ascending aorta (N/m^2^)	0.9 ± 0.3	0.4 ± 0.1	**0.01**
Proximal aortic arch (N/m^2^)	0.9 ± 0.5	0.4 ± 0.1	ns
Distal aortic arch (N/m^2^)	0.6 ± 0.2	0.5 ± 0.2	**0.037**
Descending aorta (N/m^2^)	0.6 ± 0.3	0.5 ± 0.2	ns

Values represent mean ± SD. *P* < 0.05 indicates a statistically significant difference. Significant values are in bold

### Segmental aortic wall shear stress

Segmental WSS analyses along the aortic circumference are illustrated in Fig. 5b. The highest segmental WSS was observed in the mid-ascending AAo and the proximal aortic arch before aortic valve repair.

In the mid-ascending AAo, consistent with the regions of eccentric flow, segmental WSS before aortic valve repair was most pronounced at the anterior, right-anterior, and right aortic wall. After surgery, WSS was significantly reduced in the A, RA, R, P, L, and LA segments.

In the proximal aortic arch, segmental WSS magnitude and asymmetry was most pronounced before aortic valve repair at the anterior and left-anterior aortic wall. After aortic valve repair, WSS was significantly reduced in the A, R, RP, P, LP, L, and LA segments.

At the level of the distal aortic arch, aortic valve repair significantly reduced segmental WSS at the A, RA, and R segments. At the level of the DAo, aortic valve repair significantly reduced segmental WSS at the LA and A segments. At the level of the sinotubular junction, segmental WSS was lowest and without significant changes after aortic valve repair.

## Discussion

This study demonstrated the feasibility of 4D flow CMR to evaluate the hemodynamic changes of aortic valve repair in patients with BAV and UAV. 4D flow CMR-derived angiograms and the ability to quantify abnormal flow parameters in the thoracic aorta allowed for both qualitative and quantitative assessment of the hemodynamic changes after surgical reconstruction of aortic root geometry. Our study revealed that the extent of secondary flow patterns, such as vortices and helices, flow eccentricity, as well as global and regional WSS were all significantly reduced in the ascending aorta after aortic valve repair.

Previous studies demonstrated that 4D flow CMR allows visualization of pronounced helical and vortical flow formations in the AAo in untreated patients with adult congenital heart disease [18, 40]. Such secondary flow patterns are caused by the eccentric outflow jet due to the asymmetry of the aortic valve. Bissel et al. [15] and Mahadevia et al. [26] showed that patients with BAV and L/R cusp fusion had highly eccentric outflow jets directed towards the right-anterior wall of the AAo before aortic valve repair. In our patients, the observed flow eccentricity resulted in localized elevated WSS in the mid-ascending AAo and the proximal aortic arch before aortic valve repair, which is in line with results of previous 4D flow CMR studies [21, 22].

Our study focused on changes in systolic outflow after aortic valve repair, as quantified by the extent of systolic peak velocity, aortic regurgitation, secondary flow patterns, flow displacement, and WSS. 4D flow CMR revealed a significantly decreased aortic valve regurgitation as well as reduced flow displacement and regional WSS after aortic valve repair. These 4D flow CMR-derived findings indicate an improved competence of the aortic valve and more symmetrical valve anatomy, thus success of the surgical procedure to normalize flow hemodynamics.

In our study, the recreation of the optimal aortic root geometry, including reduction of basal ring diameter and restoration of effective cusp height had two important hemodynamic effects as demonstrated by 4D flow CMR: First, reduced regurgitation, which consecutively reduces the stroke volume, and secondly, a more centralized outflow due the improved symmetry of the aortic valve. Corresponding to this postoperative hemodynamic improvement, a significant reduction of WSS was observed in the mid-ascending AAo, as well as in the proximal and distal aortic arch. A significant reduction of flow displacement was observed only in the mid-ascending AAo. This discrepancy may be explained by the fact that WSS is not only caused by local eccentric flow jets, but also by the stroke volume, which was significantly reduced after surgical aortic valve repair, thus reducing WSS.

Patients with highest flow displacement and highest WSS in the mid-ascending AAo before aortic valve repair showed the greatest changes in blood flow hemodynamics and were among the patients with lowest flow displacement and lowest WSS after aortic valve repair. This may be explained by the fact that patients with the highest flow displacement had the most asymmetric aortic valve geometry and the most severe aortic regurgitation. These patients had the greatest hemodynamic benefit from symmetrical aortic valve rearrangement correction of symptomatic aortic regurgitation.

Two patients showed an increase in circumferential WSS in the mid-ascending aorta after isolated aortic valve repair with either just a relatively small improvement or even a small increase in flow displacement. The first patient developed severe recurrent aortic regurgitation 6 weeks after surgery and underwent a redo aortic valve repair 2 months after the initial surgery. A tearing of the fused cusp at the commissural level was found intraoperatively. The second patient had an uneventful postoperative course with an excellent echocardiographic result and no residual aortic regurgitation.

Our study has potentially important clinical implications. Adult congenital heart disease, namely BAV and UAV, is associated with aortopathy and an increased risk for aortic dissection, even after aortic valve repair [5, 8]. It remains unclear, how a modified, more physiologic flow after bicuspid aortic valve repair affects the long-term outcome of aortopathy. In this context, 4D flow CMR is a comprehensive tool to monitor not only aortic valve competence but also hemodynamic changes in the thoracic aorta. Particularly 4D flow CMR-derived WSS and flow eccentricity may provide insights into the mechanisms involved in aortopathy formation.

Several studies have successfully investigated 4D flow CMR for the evaluation of hemodynamic changes after aortic valve replacement surgery [28, 43]. However, none of these studies has assessed the impact of the modified transvalvular flow patterns on the progression of aortopathy in prospective longitudinal studies. The ability of 4D flow CMR to illustrate non-physiologic blood flow patterns and measure hemodynamic parameters beyond standard metrics may allow to guide physicians towards an individualized clinical decision approach regarding aortic root replacement in patients with increased risk for progression of aortopathy after aortic valve repair or replacement.

However, we are well aware that our results represent only the first step toward long term studies with outcome metrics. Future prospective and longitudinal studies are needed to assess whether post-operative 4D flow CMR can predict the progression of aortopathy and the risk of late aortic complications as well as patient outcomes.

Our study has several limitations. First, a limited study sample size prevented us from further post-hoc subanalysis and comparison of different BAV morphologies (Sievers type 0, type 1, and type 2) and UAV morphology as well as comparison of patients with and without aortic root replacement. Future 4D flow CMR studies with a larger sample size are needed to compare the surgical impact on blood flow dynamics between patients with these different valvular morphotypes.

Second, a limited spatial resolution of our 4D flow CMR may result in underestimation of WSS [44, 45]. However, our main focus was the comparison of 4D flow CMR-derived parameters before and after aortic valve repair and all scans were performed on the same CMR unit with identical scan parameters. Therefore, relative differences in WSS before and after aortic valve repair remain reliable results.

The third limitation is the manual positioning of 2D analysis planes. We tried to minimize this potential bias by adhering to defined anatomical landmarks for positioning of the analysis planes. Still, 2D analyses may result in limited coverage and distorted quantification of complex WSS distribution when compared to more advanced 3D WSS quantification techniques [20, 27, 39]. However, we do not have access to these advanced and dedicated analyses techniques. Instead, we aimed to validate the clinical utility of currently available 4D flow CMR analyses techniques in the setting of adult congenital heart disease. Therefore, we focused on analyses methods that are established in our department and widely accepted in the 4D flow CMR community, and therefore also more likely available to other clinical sites.

## Conclusions

4D flow CMR allows assessment of the impact of aortic valve repair on changes in blood flow dynamics in patients with bicuspid aortic valve disease. Further prospective studies are required to determine whether 4D flow CMR can play an important clinical role, such as predicting progression of aortopathy and long-term risk of late aortic complications as well as patient outcomes.

## Data Availability

The datasets used and/or analysed during the current study available from the corresponding author on reasonable request.

## Abbreviations

2D: Two dimensional
4D: Four dimensional
A: Anterior
AAo: Ascending aorta
AA: Aortic arch
BAV: Bicuspid aortic valve
bSSFP: Balanced steady state free precession
CMR: Cardiovascular magnetic resonance
DAo: Descending aorta
distAA: Distal aortic arch
ECG: Electrocardiogram
EDV: End-diastolic volume
EF: Ejection fraction
ESV: End-systolic volume
L: Left
LA: Left-anterior
LP: Left-posterior
LV: Left ventricle/left ventricular
midAAo: Mid-ascending aorta
P: Posterior
proxAA: Proximal aortic arch
R: Right
RA: Right-anterior
RP: Right-posterior
STJ: Sinotubular junction
UAV: Unicuspid aortic valve
WSS: Wall shear stress
